# Activation of Antioxidant and Proteolytic Pathways in the Nigrostriatal Dopaminergic System After 3,4-Methylenedioxymethamphetamine Administration: Sex-Related Differences

**DOI:** 10.3389/fphar.2021.713486

**Published:** 2021-08-27

**Authors:** Giulia Costa, Francesca Felicia Caputi, Marcello Serra, Nicola Simola, Laura Rullo, Serena Stamatakos, Fabrizio Sanna, Marc Germain, Maria-Grazia Martinoli, Sanzio Candeletti, Micaela Morelli, Patrizia Romualdi

**Affiliations:** ^1^ Department of Biomedical Sciences, Section of Neuroscience, University of Cagliari, Cagliari, Italy; ^2^ Department of Pharmacy and Biotechnology, Alma Mater Studiorum-University of Bologna, Bologna, Italy; ^3^ Department of Medical Biology, Université du Québec à Trois-Rivières, Trois-Rivières, QC, Canada; ^4^ CERMO-FC UQAM, Québec, QC, Canada; ^5^ Department of Psychiatry and Neuroscience, Université Laval and CHU Research Center, Québec, QC, Canada; ^6^ National Research Council of Italy, Neuroscience Institute, Cagliari, Italy

**Keywords:** gluthatione peroxidase, hyperthermia, superoxide dismutase, tyrosine hydroxylase, ubiquitin-proteasome system

## Abstract

3,4-Methylenedioxymethamphetamine (MDMA, “ecstasy”) is an amphetamine-related drug that may damage the dopaminergic nigrostriatal system. To investigate the mechanisms that sustain this toxic effect and ascertain their sex-dependence, we evaluated in the nigrostriatal system of MDMA-treated (4 × 20 mg/kg, 2 h apart) male and female mice the activity of superoxide dismutase (SOD), the gene expression of SOD type 1 and 2, together with SOD1/2 co-localization with tyrosine hydroxylase (TH)-positive neurons. In the same mice and brain areas, activity of glutathione peroxidase (GPx) and of β2/β5 subunits of the ubiquitin-proteasome system (UPS) were also evaluated. After MDMA, SOD1 increased in striatal TH-positive terminals, but not nigral neurons, of males and females, while SOD2 increased in striatal TH-positive terminals and nigral neurons of males only. Moreover, after MDMA, SOD1 gene expression increased in the midbrain of males and females, whereas SOD2 increased only in males. Finally, MDMA increased the SOD activity in the midbrain of females, without affecting GPx activity, decreased the β2/β5 activities in the striatum of males and the β2 activity in the midbrain of females. These results suggest that the mechanisms of MDMA-induced neurotoxic effects are sex-dependent and dopaminergic neurons of males could be more sensitive to SOD2- and UPS-mediated toxic effects.

## Introduction

Several lines of evidence have demonstrated that 3,4-methylenedioxymethamphetamine (MDMA, or “ecstasy”) may not only elicit psychoactive effects, but also affect different neurotransmitter systems and induce neurotoxicity in experimental animals (Stone et al., 1987; Stone et al., 1989; Parrott, 2001; Easton and Marsden, 2006; Baumann et al., 2007; Gudelsky and Yamamoto, 2008). MDMA has high affinity for the serotonin transporter, and serotonergic damage elicited by MDMA has been described in several animal species and hypothesized to occur also in humans who consume this amphetamine-related drug (Stone et al., 1987; Stone et al., 1989; Parrott, 2001; Easton and Marsden, 2006; Allott and Redman, 2007; Baumann et al., 2007; Gudelsky and Yamamoto, 2008). MDMA also possesses significant affinity for the dopamine (DA) transporter (DAT) and dopaminergic (DAergic) damage elicited by MDMA has also been reported. Notably, studies in mice have demonstrated that MDMA elicits a peculiar profile of neurotoxicity in this species that involves the DAergic nigrostriatal and mesolimbic systems (Colado et al., 2001; Green et al., 2003; Cadet et al., 2007; Granado et al., 2008; Frau et al., 2013, Frau et al., 2016a; Moratalla et al., 2017; Costa et al., 2017, 2020). Thus, MDMA decreases the levels of both DA and DAT in the striatum (Iravani et al., 2000; Colado et al., 2001, 2004; Camarero et al., 2002; Green et al., 2003; Cadet et al., 2007; Costa et al., 2013, Costa et al., 2017; Halpin et al., 2014), as well as the immunoreactivity for tyrosine hydroxylase (TH), the rate-limiting enzyme for DA synthesis, in both the striatum and substantia nigra pars compacta (SNc) (Granado et al., 2008; Costa et al., 2017; Moratalla et al., 2017).

It is now well acknowledged that the neurotoxic effects of MDMA are influenced by several mechanisms that may be correlated to different factors such as sex, body temperature and also the environment where MDMA is experienced (de Win et al., 2004; Piper and Meyer, 2004; Schilt et al., 2010; Price et al., 2014; Frau et al., 2016a; Costa et al., 2019). In particular, sex is increasingly emerging as a key factor that underlies the vulnerability to neuronal death that occurs in the course of neurodegenerative diseases and in response to drugs (Della Torre and Maggi, 2017).

Previous studies from our and other laboratories have suggested that hyperthermia, which is a commonly observed noxious effect of MDMA (Green et al., 2003; Hall and Henry, 2006), may favor the neurotoxicity and/or glia activation induced by this amphetamine-related drug (Miller and O’Callaghan, 1995; Colado et al., 1998; Mechan et al., 2001; Touriño et al., 2010; Frau et al., 2013), with a mechanism that is thought to stem from increased production and release of reactive oxygen species (ROS) (Chipana et al., 2008; Sharma and Ali, 2008; Shokry et al., 2019). However, other authors have demonstrated that MDMA can induce the release of ROS and neurotoxicity in experimental animals by means of mechanisms that are independent of hyperthermia (Green et al., 2004).

In addition to hyperthermia, altered activity and/or expression of antioxidant enzymes may participate in the neurotoxic effects of MDMA. Thus, a prominent role in MDMA-induced neurotoxicity has been proposed for superoxide dismutase (SOD), which catalyzes the dismutation of the superoxide radical (O_2_^−^) produced by mitochondria into molecular oxygen (O_2_) and hydrogen peroxide (H_2_O_2_), and for glutathione peroxidase (GPx), which reduces H_2_O_2_ to H_2_O (Flohé, 1988). Indeed, it has been reported that MDMA administration was able to increase the activity of SOD in mice (Peraile et al., 2013). Moreover, others have demonstrated that the genetic overexpression of the isoform 1 of SOD (SOD1), located in the cytoplasm, protected mice from the neurotoxic damage induced by MDMA (Jayanthi et al., 1999). Collectively, these data claim for further investigation in order to clarify whether MDMA may disrupt the antioxidant barrier through modulation of these enzymes, and whether the effects of MDMA on specific antioxidant systems may be sex-dependent.

In the scenario of enzymes that may possibly be involved in the DAergic damage induced by MDMA, the ubiquitin-proteasome system (UPS) deserves consideration. Indeed, different intracellular proteins can be degraded by the UPS system, that comprises three proteolytic subunits (β1, β2, and β5) (Gallastegui and Groll, 2010). These subunits share a common proteolytic mechanism although they target different substrates with caspase-, trypsin- and chymotrypsin-like activities, respectively (Groll et al., 2005). Genetic studies established a hierarchy for the UPS subunits in relationship to their importance for cell growth, identifying the β5 chymotrypsin-like activity as the most important (Wolf and Hilt, 2004). In this frame, it has been reported that protein degradation is fundamental to counteract oxidative stress (Goldberg, 2003; Jung and Grune, 2008). Indeed, different intracellular proteins can be degraded by the UPS system and it is well known that proteasome dysfunction can be associated with an increase of oxidative products and subsequent protein aggregation (Aiken et al., 2011). Interestingly, different drugs of abuse, such as opioid agonists (Caputi et al., 2019), ethanol (Pla et al., 2014), nicotine (Rezvani et al., 2007), cocaine (Ren et al., 2013), amphetamine and amphetamine-related drugs such as methamphetamine (Fornai et al., 2004; Iwazaki et al., 2006; Lin et al., 2012), have been shown to produce dysfunctions in the UPS. As of today, only one study performed in adult male C57Bl/6J mice has explored the effects of MDMA on UPS, by demonstrating the formation of ubiquitin-positive inclusions in the cytoplasm of DAergic neurons in the SNc after the administration of MDMA (Fornai et al., 2004b).

Based on these considerations, we have investigated whether pro-oxidant and proteolytic mechanisms may participate in the neurotoxic effects that MDMA induces in the DAergic nigrostriatal system of mice and whether these proposed mechanisms of toxicity may be influenced by sex. To this end, we treated adult (12 weeks old) male and female mice with MDMA according to an administration regimen that has previously been shown to induce nigrostriatal DAergic damage (Granado et al., 2008; Frau et al., 2016a; Moratalla et al., 2017; Costa et al., 2019) and, after ascertaining the presence of hyperthermia and degeneration of DAergic nigral neurons, we evaluated in the nigrostriatal system:

1) the co-localization and/or activity of SOD1 and SOD2 antioxidant enzymes;
2) the expression of SOD1 and SOD2 genes;
3) the activity of the proteolytic subunits β2 trypsin-like and β5 chymotrypsin-like of the UPS.

## Materials and Methods

### Animals

Adult (12 weeks old, 25–30 g) male and female C57BL/6J mice (Charles River, Italy) were used in this study. Mice were housed in groups of five to six animals matched for sex and age in Plexiglas medium-sized cages (length, 42 cm; width, 24 cm; height, 15 cm) in a room under controlled temperature (21 ± 1°C), humidity (55 ± 10%) and light cycle (lights on at 8:00), with food and water available ad libitum. Experimental protocols were reviewed and approved by the Italian Ministry of Health (decree No. 529/2016-PR), in compliance with the European Council directives for care and use of experimental animals (609/86 and 63/2010) and with policies issued by the Organism for Animal Welfare (OPBA) of the University of Cagliari. Experiments were designed to minimize animal discomfort to the least possible extent and to reduce the number of animals used.

### Drugs

MDMA was synthesized and solubilized in physiological saline, as described elsewhere (Frau et al., 2013), then administered by the intraperitoneal (i.p.) route in a volume of 10 ml/kg. Mice in the control groups were treated with vehicle (i.p.), at the same time of the day as the mice that received MDMA treatment.

### MDMA Treatment

A total number of 45 mice were used in this study (five to six mice for each experimental group). Male and female mice were treated between 9:00 and 16:00 h with either MDMA (4 × 20 mg/kg, i. p. 2 h apart) or vehicle (saline solution, i. p. 4 administrations, 2 h apart). The general health of mice was evaluated every 30 min during the treatment and no signs of distress or suffering (i.e. signs of irregular respiration or tremor) were observed. The protocol and route of MDMA administration used in this study were chosen based on previous investigations that have demonstrated how the same MDMA regimen induced significant neurotoxicity in the nigrostriatal system of C57BL/6J mice (Reveron et al., 2005; Frau et al., 2016a). Although the dose of MDMA administered in this study is higher than those usually taken by recreational users (Vollenweider et al., 1998), the protocol of administration used here may be regarded as compatible to the pattern of drug intake displayed by heavy MDMA users (Jansen, 1999; McCann and Ricaurte, 2001; Parrott, 2005).

### Temperature Recording

Body temperature was measured using a rectal probe (BRET-3) digital thermometer (MicroTherma 2T Hand Held Thermometer, 2Biological Instruments, Besozzo, Varese, Italy). Temperature was recorded prior to the first administration of either MDMA or vehicle (baseline temperature), and then 1 h after each administration of either MDMA or vehicle. Temperature was recorded by holding each mouse at the base of its tail and inserting the probe past the rectum into the colon for 4–5 s, until stable rectal temperature was maintained for 3 s.

### Assessment of the Phase of Estrus Cycle in Female Mice

The phase of the estrus cycle in female mice (N = 6 mice per group) was assessed by morphological inspection of the vaginal smears collected by lavage and dyed with May Grunwald-Giemsa stain. The phase of the estrus cycle was identified according to the presence and ratio of the following three types of cells: 1) round epithelial and nucleated cells, 2) irregular shaped and cornified cells, 3) smaller and dark stained leukocytes. The following phases of the estrus cycle were identified: proestrus, prevalence of round, large and nucleated epithelial cells which could be grouped in form of layers, estrus, prevalence of large and irregular shaped cornified cells; metestrus, leucocytes, epithelial and cornified cells in equal ratio; diestrus, prevalence of leukocytes (Contini et al., 2018). Stained vaginal smears were blindly inspected by two independent observers, in order to avoid possible misinterpretations of the results.

### Brain Tissue Collection

Two hours after the completion of pharmacological treatments, all mice were treated with equithesin (pentobarbital 0.97 g, MgSO4 2.1 g, chloral hydrate 4.25 g, propylene glycol 42.8 ml, ethanol 90% 11.5 ml, to 100 ml with distilled water), administered in a volume of 5 ml/kg i. p., in order to induce profound anesthesia and minimize suffering before sacrifice.

Mice used for immunohistochemistry studies were sacrificed by transcardial perfusion with cold 4% paraformaldehyde in phosphate buffer (PB, 0.1 M, pH 7.4). Brains were then removed, post-fixed for 24 h, and processed according to the procedures previously described (Costa et al., 2014). Coronal brain sections were cut at 50 μm on a vibratome and stored in a cryoprotectant solution at −20°C until use. For each mouse, three sections were collected from the two brain regions analyzed at the following coordinates: from 1.34 to 0.74 mm (striatum) and from −2.92 to −3.52 mm (SNc), relative to bregma.

Mice used for biochemical assays and for quantitative real-time protein chain reaction (qRT-PCR) studies, were decapitated and the striatum (1.34 to 0.74 mm relative to bregma) and midbrain (−2.92 to −3.52 mm relative to bregma) were collected from freshly dissected brains, immediately frozen on dry ice and stored at –80°C until analysis.

All the brain areas were identified according to the mouse brain atlas of Paxinos and Franklin (2008).

The time of sacrifice was selected based on previous experiments performed in our laboratories, showing that: 1) repeated treatment with MDMA followed by sacrifice 2 h after discontinuation causes a reduction of TH-positive terminals and neurons in the nigrostriatal DAergic system of mice (Granado et al., 2008; Frau et al., 2016b); 2) treatment with MDMA induces alterations in the gene expression of the β2/β5 subunits of the UPS that are evident as early as 2 h after administration (Caputi et al., 2016).

### Immunohistochemistry Studies

Free-floating sections were rinsed in 0.1 M PB, blocked in a solution containing 3% normal goat serum (Sigma-Aldrich, Milan, Italy) and 0.3% Triton X-100 in 0.1 M PB at room temperature for 2 h, and incubated in the same solution with the primary antibody/ies (Table 1) for two nights at 4°C. Thereafter, the sections were rinsed three times in 0.1 M PB and incubated with the secondary antibody/ies (Table 1) in 0.1 M PB at room temperature for 2 h.

**TABLE 1:** Features and dilutions of the primary and secondary antibodies used in this study.

Antibody	Species	Type	Diluition	Supplier
TH	Rabbit	Polyclonal	1:1,000	Millipore, Temecula, CA, United States
SOD1	Mouse	Monoclonal	1:500	Millipore, Temecula, CA, United States
SOD2	Mouse	Monoclonal	1:200	Abcam, Cambridge, United Kingdom
DAT	Rat	Monoclonal	1:1,000	Millipore, Temecula, CA, United States
Anti rabbit^594^	Goat		1:500	Jackson ImmunoResearch Europe, suffolk, United Kingdom
Anti mouse^488^				
Anti rat^488^				

Afterwards, the sections were rinsed and immediately mounted onto glass slides coated with gelatin in Mowiol mounting medium. To allow visualization of cell nuclei, sections were incubated for 10 min with the nuclear marker DAPI (4′,6-diamidine-2′-phenylindole dihydrochloride, 1:10,000, Sigma-Aldrich, Milan, Italy) and then mounted onto glass slides. Omission of either the primary or secondary antibody/ies served as negative control, and yielded no labeling, as previously reported (Howat et al., 2014).

### Image Analysis

For single staining studies, images of single wavelength were obtained with an epifluorescence microscope (Axio Scope A1, Zeiss, Oberkochen, Germany) connected to a digital camera (1.4 MPixels, Infinity 3-1, Lumenera, Nepean, Canada). For double staining studies, images were obtained with a Leica TCS SP8 confocal microscope fitted with a 63x/1.40 oil objective using the optimal resolution for the wavelength used (determined by the Leica software).

For single staining, in each of the three brain sections two portions from the striatum (dorsolateral and ventromedial) and/or the whole SNc, left and right, were acquired using a ×20 objective. The ImageJ software (U.S. National Institutes of Health, United States) was used to quantify the density of immunoreacted fibers positive to DAT and/or TH in the striatum. Sections were captured in black and white 8-bit monochrome and the density of fibers was determined in fixed regions using a threshold level that was kept constant across all images. The pixels were converted into square micrometers by employing a suited calibration, in order to represent the area occupied by a specific immunoreaction product in µm^2^. Neurons positive to TH in the SNc were quantified by capturing the whole left and right areas using a ×10 objective. All the sections obtained where then evaluated with the manual particle counting option of ImageJ. The number of neurons labeled with DAPI was obtained separately for each level of the SNc. For each level of the striatum and SNc, the value obtained was first normalized with respect to vehicle, then values from different levels were averaged. No significant differences in the density of either immunoreacted fibers or neurons were found among the three coronal sections of a given area in the same mouse.

For double staining, two portions from the striatum (dorsolateral and ventromedial) and/or the whole SNc, left and right, were acquired using a ×40 objective, then images were analyzed using the ImageJ software. Quantitative analysis of co-localization of TH with SOD1 or SOD2 was conducted using the ImageJ plugin JACoP (Just Another Co-localisation Plugin) (Bolte and Cordelières, 2006). The correlation of signal intensity was calculated as Pearson correlation coefficient (Rr), as previously described (Sirabella et al., 2018).

### Activity Assay Studies

Tissue samples were homogenized in lysis buffer (150 mM NaCl, 50 mM HEPES pH 7.5, 5 mM EDTA, 2 mM ATP, 1% Triton; Sigma-Aldrich, Milan, Italy) and centrifuged at 14,000 × g at 4°C for 15 min. Homogenates were aliquoted and kept at −80°C until assays. Protein concentration was determined by using the Pierce BCA protein assay kit (Thermo Fischer Scientific, Waltham, Massachusetts, United States; CAT 23227). The antioxidant and UPS activities were analyzed using 25 μg of cell lysate proteins that were processed with the commercially available assay kits listed below.

#### SOD Activity Assay

SOD activity was determined using the SOD assay kit-WST (Sigma-Aldrich, Milan, Italy; CAT 19160-1KT-F). Briefly, the assay is based on the use of the dojindo’s highly water-soluble tetrazolium salt, WST-1 [2-(4-Iodophenyl)-3-(4-nitrophenyl)-5-(2,4-disulfophenyl)-2H-tetrazolium monosodium salt], which forms a water-soluble formazan dye upon reduction with a superoxide anion, with a reduction rate that is inversely proportional to SOD activity. The assay was carried out according to the manufacturer’s instructions. Briefly, the mouse tissue samples (20 µL) mixed with 200 µL WST working solution (1000 µl WST solution, 19000 µl of buffer solution) were allowed to react with 20 µL of enzyme working solution (15 µL of enzyme solution, 2500 µl of dilution buffer). Distilled water (ddH_2_O, 20 µL) was used as the sample substitute for blank 1 (S1) wells and 20 µL of dilution buffer as the substitute of enzyme working solution for blank 2 (S2) wells. In blank 3(S3) wells, only 20 µL of each ddH_2_O and dilution buffer were added to the 200 µL WST working solution. The reaction mix was then incubated at 37°C for 20 min. The absorbance was measured at 450 nm and the SOD activity was determined as follows:

SOD Activity (%) = {[(AbsS1−AbsS3) (Abssample−AbsS2)]/(AbsS1−Absblank)}×100

#### GPx Activity Assay

GPx activity was determined using the EnzyChromTM Glutathione peroxidase Assay Kit (BioAssay Systems, Hayward, United States; CAT EGPx-100), which directly measured the consumption of NADPH in the enzyme-coupled reactions. The assay was carried out according to the manufacturer’s instructions. The absorbance of the GPx activity was quantified at 340 nm using a microplate reader (Genios Tecan, Ramsey, Minnesota, United States). The NADPH standards were used to generate the standard curve which was used to calculate the GPx activity in the tissue samples as follows:

GPx activity (U/L) = [(ΔODSample−ΔODBackground control)/Slope (mM-1) × 4 (min)] × 1,000 × n (diluition factor).

#### β2/β5 Subunits of the UPS Activity Assay

The substrates benzyloxycarbonyl-Ala-Arg-Arg-7-amino-4-methylcoumarin (Z-ARR-AMC) and succinyl-Leu-Leu-Val-Tyr-7-amino-4-methylcoumarin (Suc-LLVY-AMC) (both purchased from Merck Millipore, Milan, Italy; CAT 539149 and 539,142 respectively) were used according to the manufacturer’s instructions to measure β2-and β5-like activities, respectively. The assay is based on the detection of the fluorophore 7-amino-4-methylcoumarin (AMC) after cleavage from the labeled substrates. The free AMC fluorescence was quantified at 380 nm excitation and 460 nm emission wavelengths using the same microplate reader cited above. An AMC standard curve was generated for reference by preparing a dilution series of AMC standard reagent in the concentration range of 0.04–12.5 μM. The assay was validated by analyzing UPS positive control incubated with the inhibitor lactacystin and two independent experiments were carried out for each tissue analyzed. Data are expressed as percentage of relative fluorescence (RFU%).

### RNA Isolation and qRT-PCR Assay

Total RNA was extracted according to the method of Chomczynski and Sacchi (1987). Each sample was subjected to DNase treatment and converted to cDNA with the GeneAmp RNA PCR kit (Life Technologies Italia, Monza, Italy; CAT N8080143), according to the manufacturer’s protocol. qRT-PCR analysis was performed on a StepOne Real-Time PCR System (Life Technologies, Monza, Italy) using the SYBR Green PCR MasterMix (Life Technologies, Monza, Italy; Cat 4309155). Relative expression of different gene transcripts was calculated by the Delta-Delta Ct (DDCt) method and converted to relative expression ratio (2-DDCt) for statistical analysis (Livak and Schmittgen, 2001). All data were normalized to the housekeeping gene glyceraldehyde-3-phosphate dehydrogenase (GAPDH). The primers used for PCR amplification were designed using Primer 3, and are here reported: GAPDH Forward 5′-AAC​TTT​GGC​ATT​GTG​GAA​GG-3′; GAPDH Reverse 5′-ACA​CAT​TGG​GGG​TAG​GAA​CA-3′; SOD1 Forward 5′-CGA​CGA​AGG​CCG​TGT​GCG​TGC​TGA​A-3′; SOD1 Reverse 5′- TGGACC ACC​AGT​GTG​CGC​CCA​ATG​A-3′; SOD2 Forward 5′- GGG​TTG​GCT​TGG​TTT​CAA​TAA​GGA​A-3′; SOD2 Reverse 5′- AGG​TAC​TAA​GCG​TGC​TCC​CAC​ACA​T-3′.

### Data Collection, Analysis and Statistics

Statistical analysis was performed with Statistica (StatSoft, Tulsa, OK, United States) and Graph Pad Prism (GraphPad, La Jolla, CA, United States). Normality of data was assessed by the D'Agostino-Pearson test and no dataset deviated from normal distribution. Regarding the evaluation of estrus phases, the presence of differences between MDMA- and vehicle-treated female mice was assessed by means of Fisher’s exact test. Regarding the other evaluations performed in this study the presence of differences between MDMA- and vehicle-treated male and female mice was assessed by means of two-way (sex × MDMA treatment) ANOVA for immunohistochemistry, activity assays and qRT-PCR studies, and by means of three-way (sex × MDMA treatment × number of MDMA administrations) ANOVA for body temperature recordings. All ANOVA analyses were followed by Newman-Keuls post-hoc test. Significance was always set at *p* < 0.05. Results were expressed as mean ± S.E.M. for every analysis performed. No randomization was performed to allocate mice to the different experimental groups and no exclusion criteria were used, resulting in the inclusion of all mice. Assessment of the phase of estrus cycle in female mice, analysis of images from immunohistochemistry, activity assay and gene expression studies were all performed by six different experimenters blinded to the experimental groups. We did not carry out a power analysis, since we relied on our previous studies that employed the same experimental protocol to pre-define the group sizes.

## Results

Adult (12 weeks old) male and female mice were treated with MDMA (4 × 20 mg/kg, i. p. 2 h apart) or vehicle (saline solution, i. p. 4 administrations, 2 h apart). Temperature and estrus cycle were evaluated before and/or during the administration of either MDMA or vehicle. Two hours after the last administration mice were sacrificed and processed for markers of neurodegeneration, oxidative stress and protein degradation in the striatum and SNc.

### Body Temperature

Treatment with MDMA increased body temperature since the first administration, although this effect reached statistical significance starting from the second administration. Three-way ANOVA revealed a significant effect of MDMA treatment (F1,20 = 96.14, *p* < 0.001) and number of MDMA administrations (F4,80 = 21.45, *p* < 0.001). Moreover, a significant interaction MDMA treatment × number of MDMA administration (F4,80 = 30.51, *p* < 0.001) was observed. In both male and female mice, Newman-Keuls post-hoc test revealed that MDMA administration increased body temperature after the second, third and fourth administration (*p* < 0.001 for all comparisons), compared with the respective groups of vehicle-treated mice (Table 2).

**TABLE 2:** Effects of treatment with MDMA on body temperature in mice. Adult male and female mice (N = 6 per group) were treated with either MDMA (4 × 20 mg/kg, i.p., 2 h apart) or vehicle (saline solution, i.p.). Body temperature values are reported as mean ± S.E.M. ***p < 0.001 compared with the respective group of vehicle-treated mice by Newman-Keuls post-hoc test.

		Rectal temperature (°C)				
		Basal	1^st^ adm	2^nd^ adm	3^rd^ adm	4^th^ adm
Male	Vehicle	37.2 ± 0.19	37.1 ± 0.18	36.8 ± 0.21	36.9 ± 0.17	36.8 ± 0.17
	MDMA	37.1 ± 0.17	37.4 ± 0.16	38.9 ± 0.19***	38.8 ± 0.18***	38.75 ± 0.21***
Female	Vehicle	37.1 ± 0.12	37.1 ± 0.15	37.3 ± 0.12	37.1 ± 0.10	37.1 ± 0.06
	MDMA	37.1 ± 0.21	37.8 ± 0.15	38.82 ± 0.23***	38.9 ± 0.29***	39.0 ± 0.43***

### Estrus Cycle in Female Mice

All female mice were in the metestrus/diestrus phase, except for two mice in the vehicle group that were in the proestrus phase (*p* = 0.45).

### Markers of Neurodegeneration in the Striatum and SNc

#### DAT Immunoreactivity in the Striatum

Treatment with MDMA modified the immunoreactivity for DAT in both male and female mice. Two-way ANOVA revealed significant effects of MDMA treatment (F1,20 = 60.12, *p* < 0.001) and sex (F1,20 = 5.92, *p* < 0.05). In both male and female mice, Newman-Keuls post-hoc test revealed that the density of immunoreacted fibers positive for DAT was reduced in the MDMA-treated groups compared with the respective vehicle-treated groups (*p* < 0.001 for both male and female mice, Table 3).

**TABLE 3:** Effects of treatment with MDMA on the immunoreactivity for the dopamine transporter (DAT) in the striatum and on the immunoreactivity for tyrosine hydroxylase (TH) in the striatum and substantia nigra pars compacta (SNc). Adult male and female mice were treated with either MDMA (4 × 20 mg/kg, i.p., 2 h apart) or vehicle (saline solution, i.p.). N = 5-6 mice per group. Values are reported as mean ± S.E.M. For DAT and TH in the striatum, data are expressed as mean density values. For TH in SNc the number of TH-positive neurons is reported. ***p < 0.001 compared with the respective groups of vehicle-treated mice by Newman-Keuls post-hoc test.

		Markers of neurotoxicity		
		DAT striatum	TH striatum	TH SNc
Male	Vehicle	194.7 ± 1.84	197.2 ± 2.74	178.2 ± 7.49
	MDMA	176.5 ± 3.36***	172.71 ± 4.90***	142.5 ± 5.27***
Female	Vehicle	190.2 ± 1.18	201.2 ± 1.39	187.3 ± 4.69
	MDMA	168.4 ± 3.27***	172.9 ± 3.29***	141.0 ± 6.76***

#### TH Immunoreactivity in the Striatum and SNc

In both the striatum and SNc, treatment with MDMA modified the immunoreactivity for TH. Two-way ANOVA revealed a significant effect of MDMA treatment (striatum: F1, 19 = 69.64, *p* < 0.001; SNc: F1, 20 = 44.39, *p* < 0.001). In both male and female mice, Newman-Keuls post-hoc test revealed that the density of TH-positive immunoreacted fibers in the striatum and the number of TH-positive neurons in the SNc were reduced in MDMA-treated groups, compared with the respective vehicle-treated groups (striatum and SNc: *p* < 0.001 for both males and females, Table 3).

### Co-Localization and/or Activity of Markers of Oxidative Stress in the Nigrostriatal System

#### TH + SOD1 Co-localization in the Striatum and SNc

In the striatum, treatment with MDMA modified the co-localization of SOD1 with TH-positive fibers. Two-way ANOVA revealed a significant effect of MDMA treatment (F1,20 = 20.39, *p* < 0.001).

**FIGURE 1:**
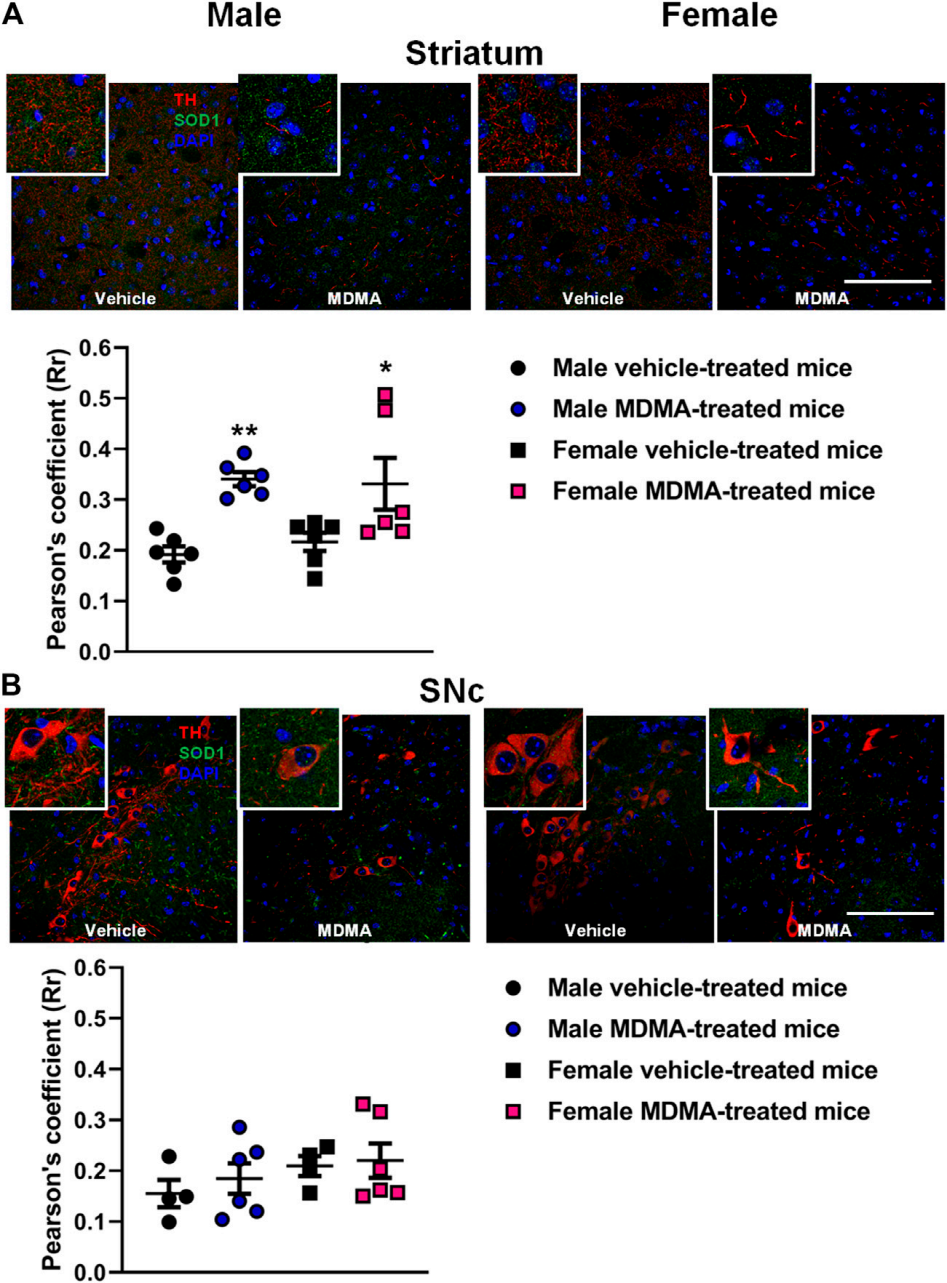
Effects of treatment with MDMA on the co-localization of superoxide dismutase 1 (SOD1) with fibers and neurons positive to tyrosine hydroxylase (TH) in the striatum **(A)** and substantia nigra pars compacta (SNc) **(B)**. Adult male and female mice were treated with either MDMA (4 × 20 mg/kg, i. p. 2 h apart) or vehicle (saline solution, i. p.). N = 5–6 mice per group. Values are reported as mean ± S.E.M. *p < 0.05, **p < 0.01 compared with the respective group of vehicle-treated mice by Newman-Keuls post-hoc test. Scale bar: 50 µm.

In both male and female mice, Newman-Keuls post-hoc test revealed that the co-localization of SOD1 with TH-positive fibers was increased in the MDMA-treated groups, compared with the respective groups of vehicle-treated mice (males: *p* < 0.01; females: *p* < 0.05, Figure 1A).

In the SNc, treatment with MDMA affected the co-localization of SOD1 with TH-positive neurons neither in male nor in female mice (Figure 1B).

#### TH + SOD2 Co-localization in the Striatum and SNc

**FIGURE 2:**
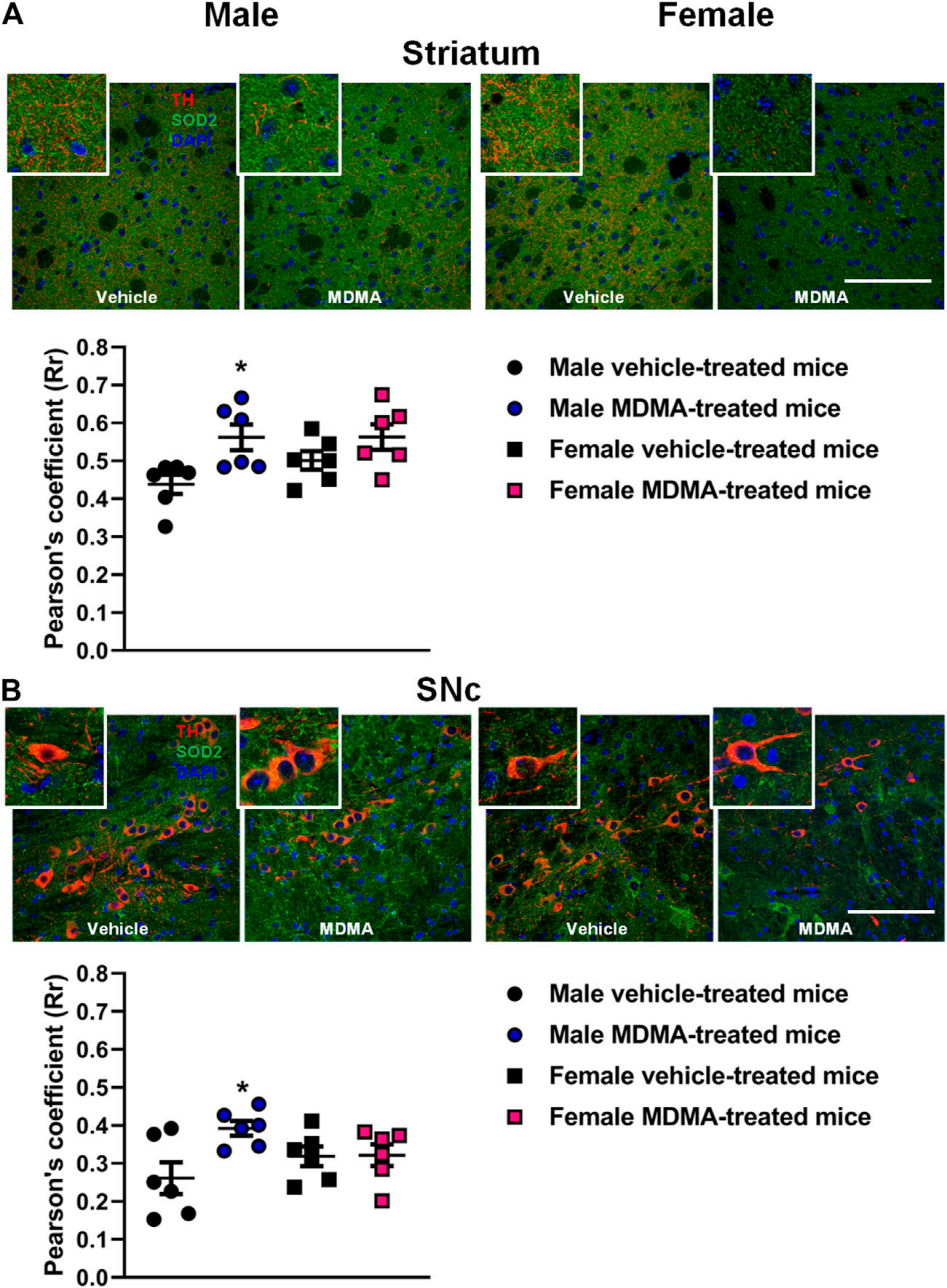
Effects of treatment with MDMA on the co-localization of superoxide dismutase 2 (SOD2) with fibers and neurons positive to tyrosine hydroxylase (TH) in the striatum **(A)** and substantia nigra pars compacta (SNc) **(B)**. Adult male and female mice were treated with either MDMA (4 × 20 mg/kg, i. p. 2 h apart) or vehicle (saline solution, i. p.). N = 6 mice per group. Values are reported as mean ± S.E.M. *p < 0.05 compared with the respective group of vehicle-treated mice by Newman-Keuls post-hoc test. Scale bar: 50 µm.

In the striatum of male mice, treatment with MDMA modified the co-localization of SOD2 with TH-positive fibers. Two-way ANOVA revealed a significant effect of MDMA treatment (F1,20 = 9.92, *p* < 0.01) and Newman-Keuls post-hoc test showed that the co-localization of SOD2 with TH-positive fibers was increased in MDMA-treated male mice compared with vehicle-treated mice (*p* < 0.05, Figure 2A). Conversely, no modifications in the co-localization of SOD2 with TH-positive fibers were observed in female mice (Figure 2A).

In the SNc of male mice, treatment with MDMA modified the co-localization of SOD2 with TH-positive neurons. Two-way ANOVA revealed a significant effect of MDMA treatment (F1,20 = 5, *p* < 0.05), as well as a significant interaction MDMA treatment × sex (F1,20 = 4.58, *p* < 0.05). Newman-Keuls post-hoc test revealed that the co-localization of SOD2 with TH-positive neurons was increased in the SNc of MDMA-treated male mice compared with vehicle-treated mice (*p* < 0.05, Figure 2B). Conversely, no modifications in the co-localization of SOD2 with TH-positive neurons were observed in the SNc of female mice (Figure 2B).

#### SOD Activity in the Striatum and Midbrain

**FIGURE 3:**
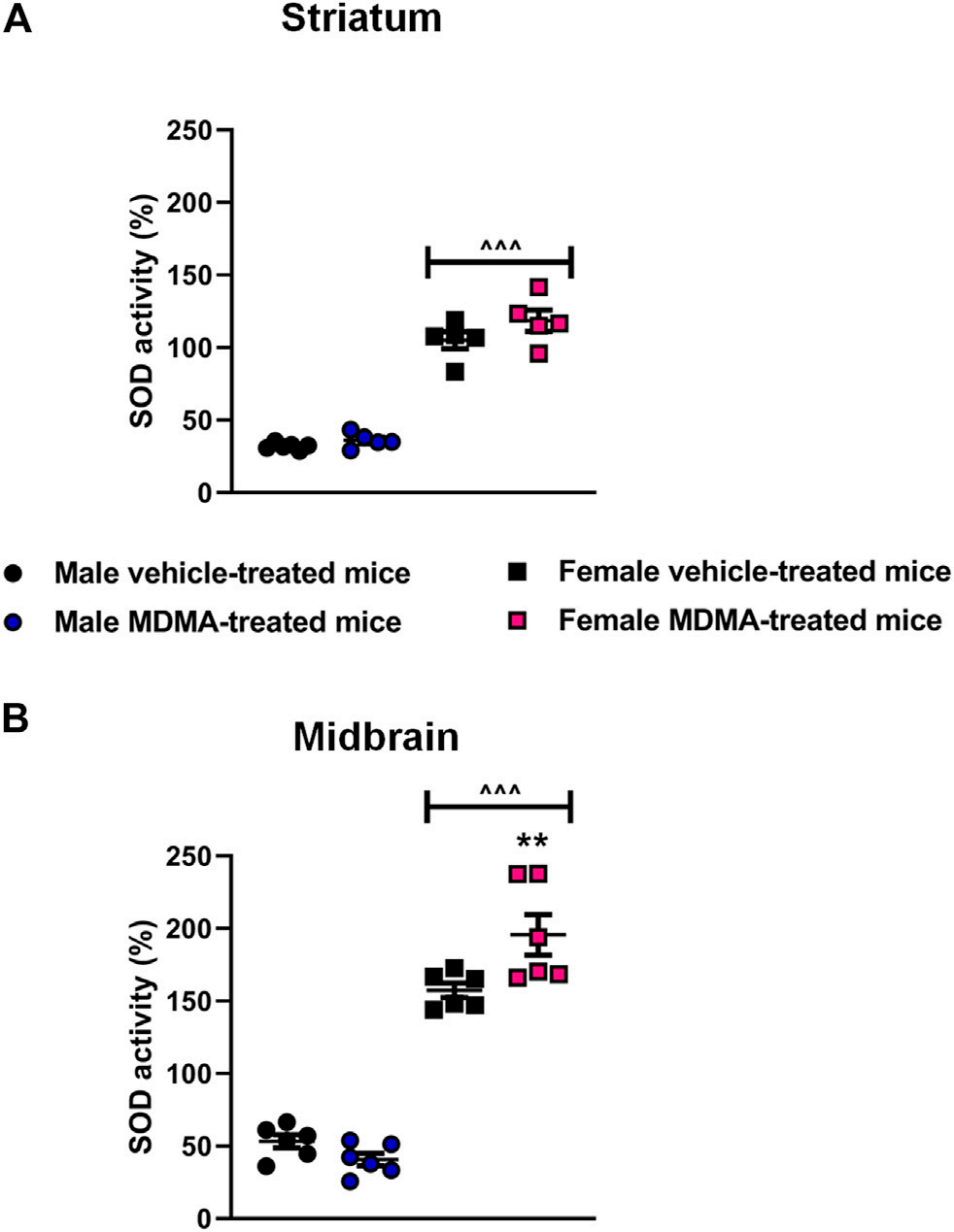
Effects of treatment with MDMA on the activity of superoxide dismutase (SOD) in the striatum **(A)** and midbrain **(B)**. Adult male and female mice were treated with either MDMA (4 × 20 mg/kg, i. p. 2 h apart) or vehicle (saline solution, i. p.). N = 5–6 mice per group. Values are reported as mean ± S.E.M. **p < 0.01 compared with the respective group of vehicle-treated mice; ^∧^^∧^^∧^ *p* < 0.001 compared with the respective groups of male mice, by Newman-Keuls post-hoc test.

In the striatum, sex-dependent modifications in SOD activity were observed. Two-way ANOVA revealed a significant effect of sex (F1,17 = 280.55, *p* < 0.001) and Newman-Keuls post-hoc test revealed a higher SOD activity in female mice, irrespective of treatment, compared with the respective groups of male mice (*p* < 0.001 for both vehicle and MDMA-treated mice, Figure 3A).

In the midbrain, sex-dependent modifications in SOD activity were observed. Two-way ANOVA revealed a significant effect of sex (F1,20 = 260.58, *p* < 0.001), as well as a significant interaction MDMA treatment × sex (F1,20 = 10.06, *p* < 0.01). Newman-Keuls post-hoc test revealed that treatment with MDMA increased SOD activity in female mice, compared with the respective group of vehicle-treated mice (*p* < 0.01, Figure 3B) as well as with the respective group of male mice (*p* < 0.001, Figure 3B). Moreover, SOD activity in vehicle-treated female mice was higher compared with that of vehicle-treated male mice (*p* < 0.001, Figure 3B).

#### SOD1 and SOD2 Gene Expression in the Striatum and Midbrain

**FIGURE 4:**
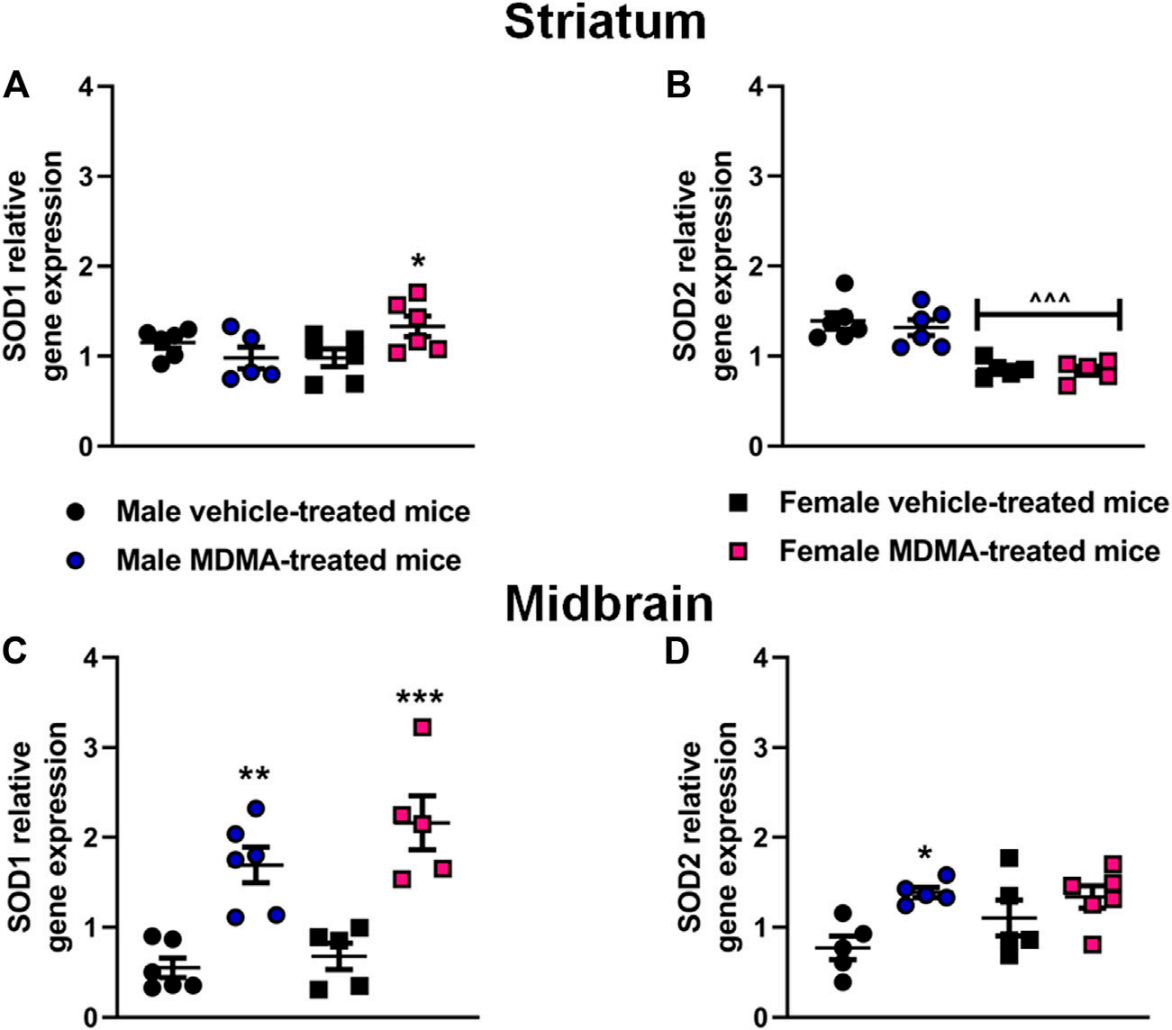
Effects of treatment with MDMA on superoxide dismutase 1 (SOD1) and superoxide dismutase 2 (SOD2) gene expression in the striatum **(A,B)** and midbrain **(C,D)**. Adult male and female mice were treated with either MDMA (4 × 20 mg/kg, i. p. 2 h apart) or vehicle (saline solution, i. p.). N = 5–6 mice per group. Values are reported as mean ± S.E.M. *p < 0.05, **p < 0.01 and ***p < 0.001, compared with the respective groups of vehicle-treated mice; ^∧^^∧^^∧^ *p* < 0.001 compared with the respective groups of male mice, by Newman-Keuls post-hoc test.

In the striatum, sex-dependent modifications in the expression of the SOD1 and SOD2 genes were observed. Two-way ANOVA for SOD1 gene expression revealed a significant interaction MDMA treatment × sex (F1,19 = 6.73, *p* < 0.05). Two-way ANOVA for SOD2 gene expression indicated a significant effect of sex (F1,18 = 44.53, *p* < 0.001). Newman-Keuls post-hoc test revealed that in female mice treatment with MDMA induced a significant up-regulation of SOD1 gene expression, compared with treatment with vehicle (*p* < 0.05, Figure 4A). Moreover, in female mice a decreased SOD2 gene expression was observed irrespective of treatment, compared with male mice (*p* < 0.001 for both vehicle and MDMA-treated mice, Figure 4B). Finally, Newman-Keuls post-hoc test disclosed that treatment with MDMA did not modify the expression of SOD1 and SOD2 genes in the striatum of male mice (Figures 4A,B).

In the midbrain, significant modifications in the expression of the SOD1 and SOD2 genes were observed in both male and female mice. Two-way ANOVA revealed a significant effect of MDMA treatment for SOD1 (F1,18 = 45.26, *p* < 0.001) and SOD2 (F1,17 = 9.61, *p* < 0.05) gene expression. In both male and female mice, Newman-Keuls post-hoc test revealed that treatment with MDMA induced a significant up-regulation of SOD1 gene expression, compared with the respective groups of vehicle-treated mice (males: *p* < 0.01; females: *p* < 0.001, Figure 4C). Moreover, in male mice Newman-Keuls post-hoc test revealed that MDMA induced a significant up-regulation of SOD2 gene expression, compared with treatment with vehicle (*p* < 0.05, Figure 4D).

#### GPx Activity in the Striatum and Midbrain

**FIGURE 5:**
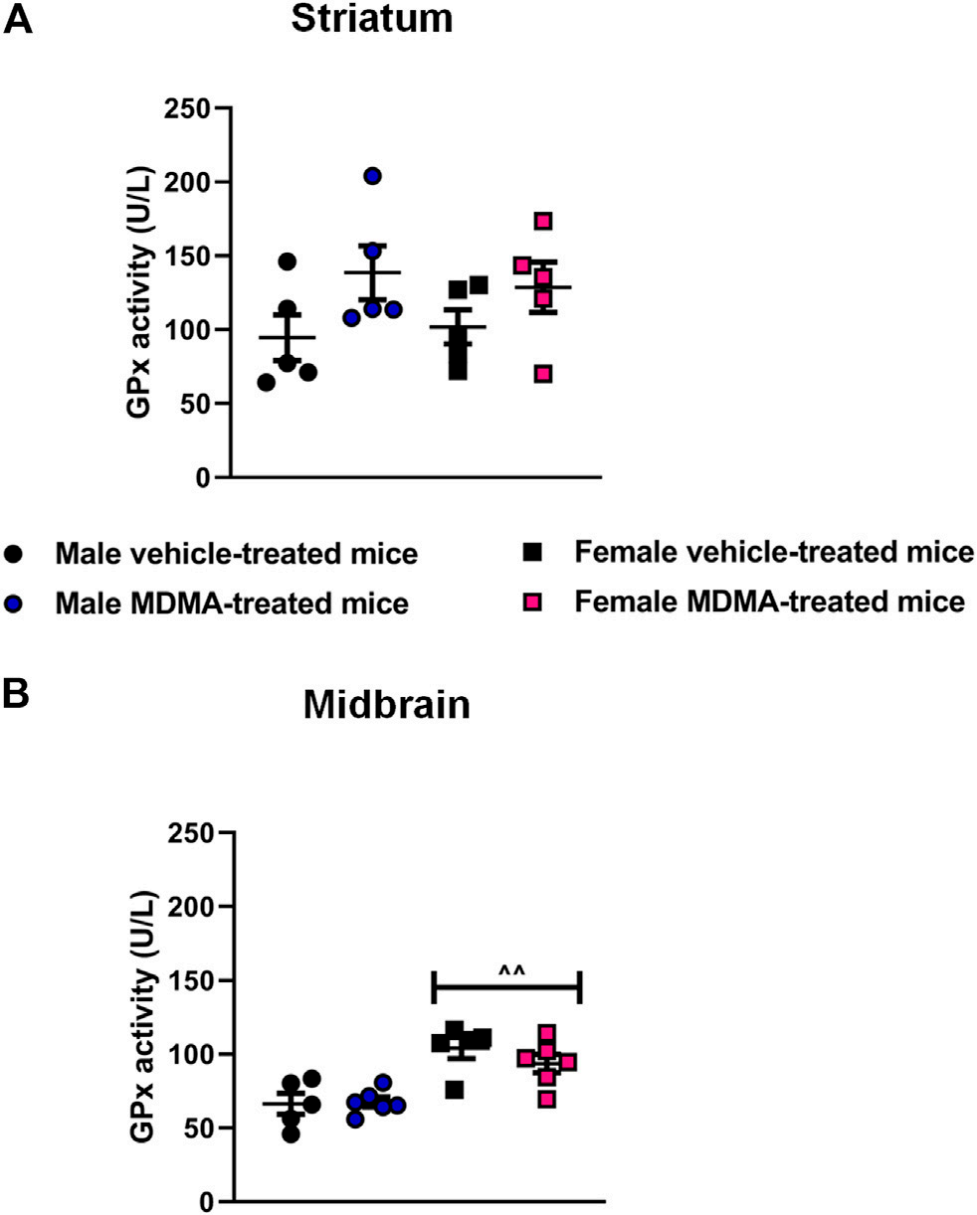
Effects of treatment with MDMA on the glutathione peroxidase (GPx) activity in the striatum **(A)** and midbrain **(B)**. Adult male and female mice were treated with either MDMA (4 × 20 mg/kg, i. p. 2 h apart) or vehicle (saline solution, i. p.). N = 5–6 mice per group. Values are reported as mean ± S.E.M. ^∧^^∧^ *p* < 0.01 compared with the respective groups of male mice, by Newman-Keuls post-hoc test.

In the striatum, no significant differences in GPx activity were observed among experimental groups. Two-way ANOVA revealed a significant effect of MDMA treatment (F1,16 = 5.04 *p* < 0.05), but Newman-Keuls post-hoc test showed no significant differences between MDMA- and vehicle-treated mice, either males or females. Nevertheless, trends towards increased GPx activity were observed in MDMA-treated male and female mice, compared with vehicle-treated mice (Figure 5A).

In the midbrain, sex-dependent modifications in GPx activity were not observed after MDMA treatment. Two-way ANOVA revealed a significant effect of sex (F1,18 = 28.01, *p* < 0.001), and Newman-Keuls post-hoc test revealed an increased GPx activity in female mice irrespective of treatment, compared with the respective groups of male mice (*p* < 0.01 for both vehicle and MDMA-treated mice, Figure 5B).

### UPS Activity in the Striatum and Midbrain

In the striatum, sex-dependent modifications were observed in both the β2 trypsin-like and the β5 chymotrypsin-like activity. Two-way ANOVA revealed a significant effect of sex (β2: F1,18 = 78.87, *p* < 0.001; β5: F1,16 = 21.20, *p* < 0.001), as well as a significant interaction MDMA treatment × sex (β2: F1,18 = 5.29, *p* < 0.05; β5: F1,16 = 11.21, *p* < 0.01).

**FIGURE 6:**
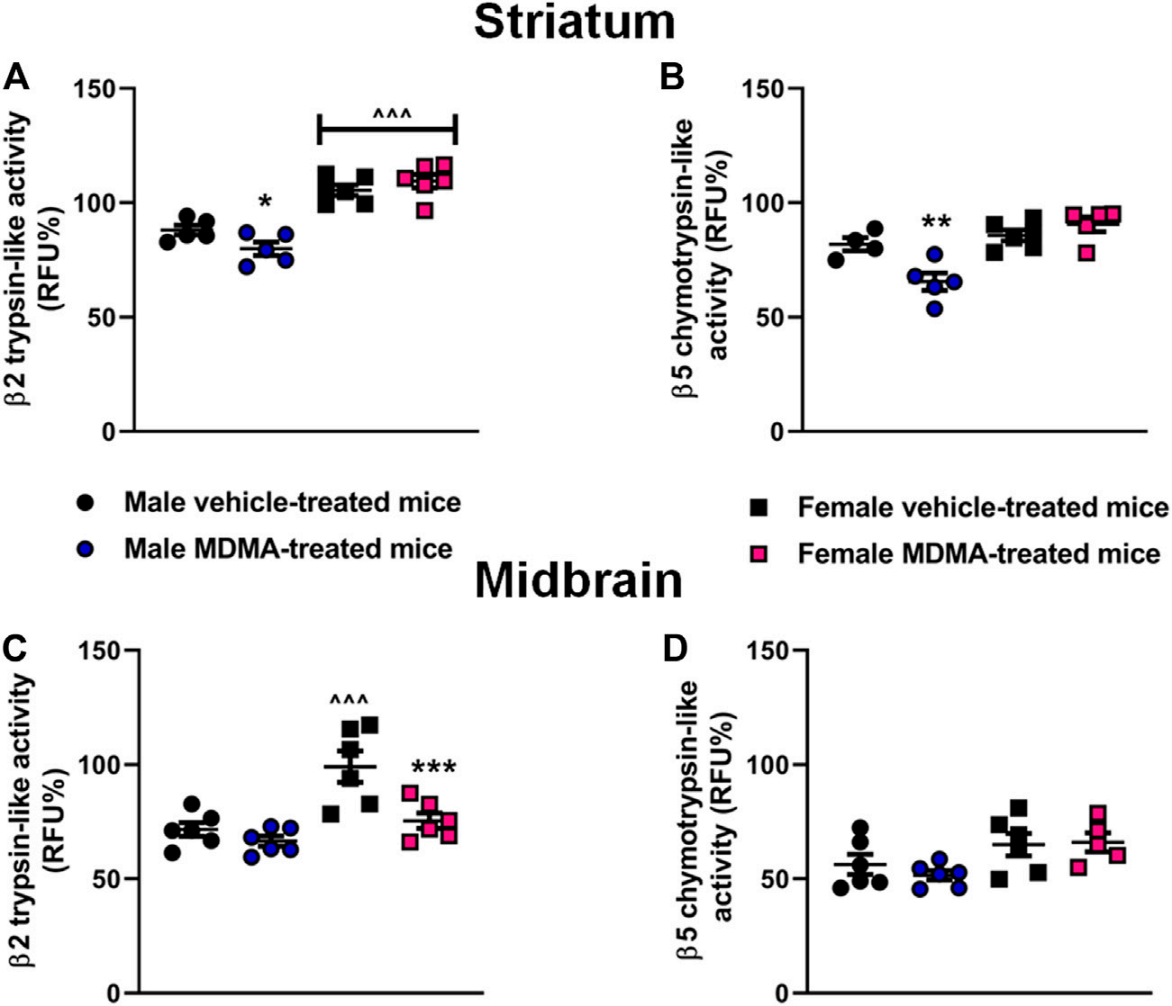
Effects of treatment with MDMA on β2 trypsin-like activity and β5 chymotrypsin-like activity in the striatum **(A,B)** and midbrain **(C,D)**. Adult male and female mice were treated with either MDMA (4 × 20 mg/kg, i. p. 2 h apart) or vehicle (saline solution, i. p.). N = 5–6 mice per group. Values are reported as mean ± S.E.M. *p < 0.05, **p < 0.01 and ***p < 0.001, compared with the respective group of vehicle-treated mice; ^∧^^∧^^∧^ *p* < 0.001 compared with the respective group of male mice, by Newman-Keuls post-hoc test.

Newman-Keuls post-hoc test revealed that treatment with MDMA decreased β2 trypsin-like and β5 chymotrypsin-like activity in male mice, compared with treatment with vehicle (β2: *p* < 0.05; β5: *p* < 0.01, Figures 6A,B). Moreover, Newman-Keuls post-hoc test revealed that female mice had higher β2 trypsin-like activity irrespective of treatment, compared with the respective groups of male mice (*p* < 0.001 for both vehicle and MDMA-treated mice, Figure 6A).

In the midbrain, MDMA administration modified the β2 trypsin-like activity in female mice. Two-way ANOVA for β2 trypsin-like activity revealed a significant effect of MDMA treatment (F1,20 = 11.59, *p* < 0.01), as well as a significant interaction MDMA treatment × sex (F1,20 = 4.77, *p* < 0.05) Moreover, two-way ANOVA revealed a significant effect of sex for both β2 trypsin-like and β5 chymotrypsin-like activities (β2: F1,20 = 18.48, *p* < 0.001; β5: F1,19 = 8.14, *p* < 0.05). Newman-Keuls post-hoc test revealed that in female mice treatment with MDMA decreased β2 trypsin-like activity, compared with treatment with vehicle (*p* < 0.001, Figure 6C). Finally, Newman-Keuls post-hoc test revealed no modifications in β2 trypsin-like activity in male mice, and no changes in β5 chymotrypsin-like activity in both male and female mice, irrespective of treatment (Figures 6C,D).

## Discussion

The present study adds important findings to the current evidence on the neurotoxic effects of MDMA (Cadet et al., 1994; Miller and O’Callaghan, 1995; Reveron et al., 2005; Touriño et al., 2010; Frau et al., 2016b; Costa et al., 2019), by evaluating possible mechanisms that participate in MDMA-mediated toxicity on the DAergic nigrostriatal pathway and the existence of sex differences in these mechanisms.

It has been previously proposed that hyperthermia induced by amphetamine-related drugs may increase the formation of ROS and reactive nitrogen species (Chipana et al., 2008; Sharma and Ali, 2008; Shokry et al., 2019). However, other studies have demonstrated that MDMA may increase the levels of oxidative stress by mechanisms other than the induction of hyperthermia (Jayanthi et al., 1999; Peraile et al., 2013). In order to elucidate whether modifications in endogenous antioxidant systems participate in the pro-oxidant effects of MDMA, we investigated in the nigrostriatal system of adult male and female mice the existence of co-localization between SOD1/SOD2 and a DAergic marker, as well as the activity and/or gene expression of SOD, GPx and the proteolytic subunits β2 trypsin-like and β5 chymotrypsin-like of UPS.

We first evaluated whether sex differences may exist in the hyperthermic and neurotoxic effects elicited by MDMA administration in mice. Our results showed that MDMA stimulated hyperthermia and a widespread nigrostriatal neurodegeneration, consisting in decreased immunoreactivity for DAT and TH in the striatum along with a decrease in TH-positive neurons in the SNc. These effects were observed in both male and female mice, which indicates that sex does not influence the neurotoxicity and hyperthermia elicited in adult mice by the protocol of MDMA administration applied in this study. The absence of sex differences in the MDMA-induced nigrostriatal neurotoxic effects may appear unexpected, since clinical studies have suggested that in MDMA users a greater depletion of serotonin may occur in women than in men (Pardo-Lozano et al., 2012). Moreover, adult female C57BL/6J mice treated with the same regimen of MDMA employed in this study but sacrificed 48 h after the last drug administration, did not show any modifications in the levels of TH in the striatum, probably due to TH axonal sprouting (Costa et al., 2019). Therefore, it should be considered that the mechanisms of neurotoxicity are complex and multiple factors are involved, suggesting that dysfunctions in the antioxidant system may be part of the mechanism underlying the neurotoxic effects of MDMA at the level of the dopaminergic nigrostriatal system.

Regarding female mice, we cannot rule out any effects mediated by estrogens since we observed that only two mice in the vehicle group were in the proestrus phase, whereas all the other female mice were in the metestrus/diestrus phase. Starting from the results that showed similar neurotoxic effects of MDMA in male and female mice, we evaluated whether the nigrostriatal system of male and female mice coped with the neurotoxic stimulus induced by MDMA by activating the same antioxidant systems, since earlier studies have demonstrated that sex differences may exist in the activation of neuroprotective pathways after exposure to neurotoxins (Yuan et al., 2009; Torres-Rojas and Jones, 2018).

The results of the present study demonstrate that sex differences exist in the antioxidant systems that are activated in the nigrostriatal pathway in response to the neurotoxic damage induced by MDMA. Indeed, an increased co-localization of SOD1 with TH-positive fibers was observed in the striatum of both male and female mice, whereas an increased co-localization of SOD2 with TH-positive fibers and neurons was observed in the striatum and SNc of male mice only.

It is noteworthy that the increase in SOD1 and SOD2 co-localization with TH observed in male mice did not correspond to an increase in SOD activity. As previously suggested by a study in brains of patients suffering from Alzheimer’s disease, this discrepancy has two possible explanations: first, the excess of SOD enzyme that is synthesized under conditions of high oxidative stress may be subsequently inactivated; second, the SOD enzyme could be concentrated in the site of increased oxidative stress, despite an overall reduction in its activity (Omar et al., 1999).

Finally, a different pattern of modifications involving TH + SOD1 co-localization and SOD1 gene expression was observed in the striatum and SNc/midbrain after MDMA administration. In the striatum, the co-localization of SOD1 with TH increased in both male and female mice, whereas SOD1 gene expression increased only in females. Conversely, in the SNc/midbrain of both male and female mice MDMA administration did not modify the co-localization of SOD1 with TH, but increased SOD1 gene expression. In order to explain these discrepant results, we may hypothesize that SOD1 mRNA was synthesized in the midbrain and then transported by axons to the striatal presynaptic terminals. This view may be sustained by data obtained in studies concerned with other enzymes that may protect the dopaminergic nigrostriatal system from degeneration (Millecamps and Julien, 2013), such as glucose-6-phosphate dehydrogenase (Mejìas et al., 2006), which have demonstrated that the intracellular transport of molecules along the axon is a crucial event for neuronal function and viability (Millecamps and Julien, 2013). In this regard, SOD1 should be considered as a first line of defense against oxidative stress because of its immediate ROS scavenging properties (Tsang et al., 2014). Accordingly, the increased levels of SOD1 in TH-positive fibers in the striatum and the increased SOD1 gene expression in the midbrain found after MDMA administration are not surprising. In fact, similar modifications involving SOD1 and its gene have been demonstrated also in rodents treated with drugs of abuse other than MDMA, including cocaine (Dietrich et al., 2005) and methamphetamine (Imam et al., 2001) although these adaptive changes, similarly to what observed here for MDMA, were not able to fully prevent the neuronal damage.

It is noteworthy that in male mice MDMA administration elicited a similar pattern of modifications in TH + SOD2 co-localization in the striatum and SNc, as well as in the expression of SOD2 gene in the midbrain, since all these parameters were increased. SOD2 is a key mitochondrial antioxidant and scavenging enzyme, thus we may speculate that the surge in SOD2 observed in male mice treated with MDMA may be due to the presence of high levels of O_2_^−^ radicals generated as a byproduct of the oxidative phosphorylation (Chance et al., 1979; Kudin et al., 2004), an effect that may not occur, or may occur only partially, in female mice. Indeed, there is solid evidence to suggest that elevated production of mitochondrial O2− is a contributing factor in neurodegenerative diseases such as Parkinson’s disease, which is characterized by the loss of DA neurons in the SNc and is more frequent in men than in women (Lin and Beal, 2006; Gillies et al., 2014). Moreover, in neurodegenerative diseases, mutations in SOD enzymes have been characterized and linked to the synthesis of cytotoxic protein aggregates that may influence the antioxidant function of SOD enzymes themselves (Pasinelli et al., 2004). Collectively, these results let us to hypothesize that male mice were more prone to activate endogenous antioxidant systems following MDMA administration, in particular at the mitochondrial level. However, since MDMA administration resulted in neuronal toxicity in both male and female mice, it may be possible that the activation of endogenous antioxidant systems may be not sufficient to prevent the neurotoxic effects of MDMA, and/or may itself trigger the release of cytotoxic/pro-apoptotic mediators that may, in turn, reduce neuronal viability (Orrenius et al., 2007).

In the present study we have also evaluated GPx activity, in order to verify whether the H_2_O_2_ produced by the increase in SOD1 and SOD2 was detoxified by the conversion to water, or a lack of detoxification could contribute to the nigrostriatal neurodegenerative process. We observed a general higher GPx activity in the midbrain of female mice, but no modifications in GPx activity in the striatum of male and female mice, as well as in the midbrain of male mice after MDMA exposure. These results are worth consideration because in physiological conditions elevated SOD activity is usually accompanied by an increase in the activity of GPx in a so-called “adaptive response” (Ceballos et al., 1988). Importantly, the lack of alterations in GPx activity after MDMA treatment could be explained by the evidence that the activity of this enzyme is strictly dependent on the amount of glutathione, and that the latter seems to be reduced following MDMA exposure (Puerta et al., 2010). In particular, it has been reported that even a small reduction in the levels of glutathione in the striatum can be sufficient to exacerbate the DA depletion induced by MDMA in the same region (Sanchez et al., 2003; Puerta et al., 2010). The results of the present study showed that the increase in SOD activity in the midbrain of female mice observed after MDMA administration was not associated with an increase in GPx activity. This latter finding is in line with a previous study reporting that MDMA (2 × 20 mg/kg, 3 h apart) increased the activity of SOD, but not GPx, in the striatum of NIH/Swiss mice, evaluated 1 h after MDMA administration (Peraile et al., 2013). Furthermore, in physiological conditions, GPx also reduces H_2_O_2_ derived from polyunsaturated fatty acids, counteracting the toxic effects of lipid peroxidation (Lee et al., 2020). We may speculate that in MDMA-treated female mice increased lipid peroxidation may occur, possibly contributing to neuronal degeneration. This speculation is supported by data obtained in several studies concerned with neurodegenerative diseases, where lipid peroxidation has been demonstrated to participate in the generation of neuronal damage and of pro-oxidant molecules (Peña-Bautista et al., 2019). Future studies are required to confirm this hypothesis also in the protocol of MDMA administration applied in the present study.

Finally, starting from the results obtained with SOD1 and SOD2 co-localization, activity, and gene expression, we evaluated the activity of the UPS, since all these cellular pathways are interconnected. UPS, as a highly regulated mechanism of intracellular protein degradation and turnover, mainly works to eliminate damaged proteins that may potentially contribute to the neurodegenerative process (Caputi et al., 2015, 2020). Hence, we aimed at evaluating whether, under oxidative stress conditions that may be induced by exposure to MDMA, activation of the UPS represented an additional way that neurons could use to eliminate damaged proteins (Caputi et al., 2016), and whether sex differences in this effect existed. Our results showed that MDMA administration decreased the β2 trypsin-like and the β5 chymotrypsin-like activities in the striatum only in male mice. Moreover, MDMA administration decreased the β2 trypsin-like activity in the midbrain of female mice to values similar to those observed in MDMA-treated male mice. Interestingly, the β2 trypsin-like activity in the striatum and midbrain of female mice was found to be higher, compared with that of male mice. These results are noteworthy, since they are the first to characterize the activity of the UPS subunits in basal conditions and after MDMA administration in male and female mice. Moreover, the results obtained in the striatum for both the β2 trypsin-like and the β5 chymotrypsin-like activities suggest that the UPS activity was impaired after MDMA administration in male but not in female mice. The idea that in conditions of heightened oxidative stress the UPS activity in male mice may be more affected than in female mice is supported by recent data in a SOD1 amyotrophic lateral sclerosis mouse model, that revealed an impaired UPS activity in males compared with females (Jenkins et al., 2020).

In conclusion, the results of the present study provide further evidence that MDMA administration induces neurotoxic effects in the nigrostriatal system of male and female mice, and suggest that the mechanisms underlying these effects may be, at least in part, sex-dependent. In particular, the nigrostriatal system of male mice appears to be more sensitive to mitochondria-mediated oxidative effects and UPS activity deficits, compared to the nigrostriatal system of female mice. The results obtained in this study appear of particular interest, since the possible existence of sex-related differences in the neurotoxic effects elicited by drugs of abuse is a hot topic in neuropharmacology, and since most of the studies performed so far involve male animals only. Nevertheless, mechanisms other than oxidative stress and protein degradation deficits deserve further investigation in the context of MDMA-induced neurotoxicity, in order to thoroughly characterize other sex-related differences in the noxious central effects of MDMA.

## Data Availability

The raw data supporting the conclusions of this article will be available upon reasonable request to MM.
